# Managing emotions in psychosis: Evaluation of a brief DBT‐informed skills group for individuals with psychosis in routine community services

**DOI:** 10.1111/bjc.12359

**Published:** 2022-02-07

**Authors:** Caroline Lawlor, Silia Vitoratou, James Duffy, Ben Cooper, Tanisha De Souza, Clair Le Boutillier, Ben Carter, Claire Hepworth, Suzanne Jolley

**Affiliations:** ^1^ Department of Psychology Institute of Psychiatry, Psychology and Neuroscience King’s College London London UK; ^2^ South London and Maudsley NHS Foundation Trust London UK; ^3^ Psychometrics and Measurement Lab Department of Biostatistics and Health Informatics Institute of Psychiatry, Psychology and Neuroscience King’s College London London UK; ^4^ Department of Health Service & Population Research Institute of Psychiatry, Psychology & Neuroscience London UK

**Keywords:** CBTp, dialectical behavior therapy, emotional dysregulation, group, schizophrenia

## Abstract

**Objectives:**

Individuals with psychosis report that emotion regulation (ER) difficulties are treatment priorities, yet little is known about how targeted ER interventions may help. We evaluated a new eight‐session Dialectical Behavioural Therapy (DBT)–informed skills group specifically adapted for individuals with psychosis: the Managing Emotions Group (MEG) in diverse, inner‐city community services.

**Method:**

A mixed‐method design was utilised to assess the feasibility (acceptability and potential clinical impact) of local delivery of MEG. Uptake, completion (≥50% of sessions), post‐session satisfaction ratings, and thematic analysis of qualitative feedback from 12 completers assessed acceptability. Pre–post‐intervention changes in psychological distress, self‐reported ER difficulties, and adaptive ER skill use assessed potential clinical impact.

**Results:**

Forty‐eight individuals (81% of attenders) completed the intervention (*M*_age_ = 43, 54% female) of whom 39 completed pre‐ and post‐group measures. Participants reported high satisfaction and meaningful improvements in understanding and managing emotions, with positive impact on daily life. Self‐reported psychological distress, ER difficulties, and adaptive ER skill use significantly improved, with medium‐to‐large pre‐post effects (*d* = 0.5–0.7) except lack of emotional clarity (*d* = 0.3).

**Conclusions:**

MEG was feasible and acceptable, and a future feasibility randomised controlled trial is warranted.

**Practitioner points:**

- Individuals with psychosis report that support with their emotions is a priority.
- Brief interventions for emotion regulation difficulties are acceptable to individuals with psychosis and can be feasibly delivered in a local outpatient service.
- Distress and emotion regulation difficulties and skills improved significantly from pre–post treatment for clients completing the managing emotions group.
- Further implementation and evaluation are needed to support continued refinement to meet the needs and priorities of individuals with psychosis.

## Background

Psychosis is a debilitating mental health condition, associated with significant costs to individuals, caregivers, and society (Ahlem et al., 2017; Chong et al., 2016; Jin, & Mosweu, 2017). Individuals with psychosis die ≤14.5 years earlier than the general population (Hjorthøj, Stürup, McGrath, & Nordentoft, 2017). Employment rates are low and mental health service use is considerable, contributing to estimated annual societal costs of £11.8 billion (Andrew, Knapp, McCrone, Parsonage, & Trachtenberg, 2012). Around 40% of individuals experience ongoing distressing symptoms and poor recovery despite taking medication; suicide rates far exceed general population levels (Girgis, 2020; Greenwood et al., 2018; Morgan et al., 2012). Cognitive behavioral therapy for psychosis (CBTp) is recommended by the National Institute for Health and Care Excellence (NICE, 2014) to reduce distress and improve coping and quality of life. However, three key issues limit implementation in routine practice. First, CBTp is resource‐intensive and rarely available at capacity to meet demand (Haddock et al., 2014; Schizophrenia Commission, 2012). Second, active engagement in CBTp is demanding: many individuals do not take up full therapy (Freeman et al., 2013; Holding, Gregg, & Haddock, 2016). Finally, diffuse therapy targets contribute to modest average treatment effects (Jauhar et al., 2014; NICE, 2014): intervention components targeting specific psychological processes obtain higher effects (Lincoln & Peters, 2019). Since targeted interventions are often protocolised and briefer than full CBTp, they are potentially easier to engage with and deliverable by a wider workforce, in greater volume (Jolley, 2018). Improved implementation requires widely applicable therapy targets (relevant for as many individuals as possible; few/no exclusion), efficient delivery (as few sessions as possible), and service‐relevant outcomes (e.g., recovery/reduced service use) (see Freeman et al., 2021; Freeman, Taylor, Molodynski, & Waite, 2019; Greenwood et al., 2018; Opoka & Lincoln, 2017).

### Emotions and psychosis

Emotional difficulties are a prominent feature of psychosis with important implications for recovery (Gumley, Gillham, Taylor, & Schwannauer, 2013; Wallace & Docherty, 2020). High levels of negative emotion are common and associated with worse outcomes, including more severe and distressing psychotic symptoms, poorer functioning, and reduced quality of life (Braga, Reynolds, & Siris, 2013; Hartley, Barrowclough, & Haddock, 2013; Li et al., 2020). Psychosis is also characterised by intense emotional reactions to stressful events and daily hassles (Myin‐Germeys & van Os, 2007), slow return to baseline levels of emotion (Vaessen et al., 2019), and heightened subjective emotional intensity (Kimhy et al., 2014). One process particularly relevant to understanding and treating emotional difficulties in psychosis concerns individual abilities to adaptively regulate their emotions (Bernard, Jackson, & Birchwood, 2015). Emotion regulation (ER) refers to the “processes by which individuals influence which emotions they have, when they have them, and how they experience and express these emotions” (Gross, 1998, p. 275). ER can be conceptualised as involving the application of particular *strategies* (e.g., reappraisal) to influence the experience or expression of emotions (Gross, 1998) or in terms of underlying dispositional *abilities* to understand, relate, and respond to emotions (Gratz & Roemer, 2004; Thompson, 1994). Recent accounts emphasise the complexity of the construct and suggest that abilities and strategies are distinct but interconnected facets of ER, and adaptive ER requires flexible application of strategies tailored to the context and individual goals (Dixon‐Gordon, Aldao, & De Los Reyes, 2015; Doré, Silvers, & Ochsner, 2016; Ford & Gross, 2018; Gratz, Dixon, Kiel, & Tull, 2018; Gross, 2015). One clinically relevant, empirically supported model of ER abilities and difficulties is provided by Gratz and Roemer (2004). Grounded in theory and research on the functionality of emotions (Cole, Michel, & Teti, 1994; Thompson, 1994) and the paradoxical consequences of avoiding, suppressing, or controlling emotions (see Hayes, Luoma, Bond, Masuda, & Lillis, 2006), the model emphasises adaptive responses to emotions (regardless of the quality of emotional responses). Gratz and Roemer (2004) conceptualise ER as involving (1) emotional awareness, understanding, and acceptance; (2) abilities to engage in goal‐directed behaviors and inhibit impulsive behaviors when experiencing negative emotions; (3) flexible ER strategy use tailored to context and individual goals; and (4) willingness to experience negative emotions in the pursuit of meaningful activities. Deficits in any of these areas indicate ER difficulties and potential treatment targets (Gratz & Roemer, 2004; Gratz & Tull, 2010).

### ER difficulties in psychosis as a treatment target

Several lines of research support ER difficulties as a treatment target in psychosis. First, individuals with psychosis report that developing skills and confidence in managing emotions is essential to recovery and an important, sometimes neglected, focus of psychological therapy (Greenwood et al., 2010; Holding et al., 2016; Law & Morrison, 2014; Lawlor et al., 2016). Second, accumulated evidence indicates that self‐reported ER difficulties are common. Following Gratz and Roemer’s (2004) definition, individuals with psychosis self‐report greater difficulties in identifying, understanding, and accepting emotions, engaging in goal‐directed behavior and willingness to experience negative emotions compared to non‐clinical controls (Lawlor, Hepworth, Smallwood, Carter, & Jolley, 2020) and comparable distress tolerance difficulties to those of individuals with borderline personality disorder, a client group characterised by significantly impaired distress tolerance (Bonfils & Lysaker, 2020). Furthermore, experience sampling and objective laboratory‐based ratings show greater difficulties in tolerating distress (Chiappelli et al., 2014; Nugent, Chiappelli, Rowland, Daughters, & Hong, 2014) and effectively applying ER strategies compared to non‐clinical controls (e.g., Opoka, Sundag, Riehle, & Lincoln, 2021; Perry, Henry, Nangle, & Grisham, 2012; Strauss et al., 2019). Finally, self‐reported ER difficulties are associated with worse outcomes, including more severe psychotic symptoms, increased hopelessness and lower self‐esteem, and mastery and psychosocial functioning (D'Antonio, Kahn, McKelvey, Berenbaum, & Serper, 2015; Kimhy et al., 2012; Liu, Chua, Chong, Subramaniam, & Mahendran, 2020; Perry, Henry, & Grisham, 2011). ER difficulties (awareness, acceptance, tolerance, and modification of emotions) also predict increased stress sensitivity (Lincoln, Hartmann, Köther, & Moritz, 2015), and lower emotional awareness mediates the relationship between ER skill use and psychotic symptoms in everyday life (Kimhy et al., 2020; Ludwig, Mehl, Schlier, Krkovic, & Lincoln, 2020). Taken together, these findings indicate that individuals report a range of ER difficulties and may benefit from targeted interventions.

Dialectical behavior therapy (DBT) is an empirically supported treatment developed specifically for treating emotional dysregulation (Linehan, 1993, 2015). Full‐model DBT is intensive, comprising individual therapy sessions, skill training groups, telephone coaching, and a consultation team; however, skill training groups are often delivered as a standalone treatment and may be more suitable in some settings (Gratz, Bardeen, Levy, Dixon‐Gordon, & Tull, 2015; Valentine, Smith, & Stewart, 2020). Importantly for implementation, accumulated evidence supports the efficacy of standalone DBT skills training groups (including 8‐week interventions) in improving ER difficulties and psychological wellbeing across a range of clinical settings and populations including individuals with mood disorders, borderline personality disorder, and transdiagnostic samples (see Valentine et al., 2020 for a review).

There are emerging literature to support brief emotion‐focused interventions for individuals with psychosis (see Opoka, Ludwig, & Lincoln, 2018 for a review), and two recent studies have evaluated brief DBT‐informed interventions specifically targeting ER difficulties. First, in a small case series, Silva, Maguire, McSherry, and Newman‐Taylor (2020) evaluated an 8‐session individual intervention. Six out of seven participants self‐reported improvements in overall ER difficulties and negative emotions and four reported reduced paranoia, with some effects maintained at follow‐up. Ryan et al. (2021) conducted a larger service‐based evaluation of an 8‐session DBT/CBTp—informed group for inpatients and outpatients with psychosis and reported significant improvements in overall self‐reported ER difficulties, mindfulness, and recovery, maintained at one‐month follow‐up. Psychotic symptoms reduced from pre‐intervention to follow‐up, and pre–post improvements in ER difficulties were associated with improved recovery and delusional symptoms at follow‐up. The therapeutic environment was rated highly. Both studies indicate that DBT‐informed skills training may hold promise; however, the feasibility and acceptability of implementation in an inner‐city community context (with high rates of diversity, substance use, socio‐economic deprivation and psychosis incidence) and specifically with individuals with established psychosis (as distinct from early intervention) are unknown. Information on local uptake and completion, service‐user views, and potential clinical impact is needed to ensure interventions are acceptable and address the specific priorities and needs of people with psychosis (Moritz, Berna, Jaeger, Westermann, & Nagel, 2017).

### Evaluation aims

Our objective was to evaluate whether a DBT‐informed intervention, the Managing Emotions Group (MEG), was feasible for local implementation in our community psychosis services. A DBT‐informed approach was chosen because DBT is a well‐established empirically supported treatment for ER difficulties where it is principle‐based and can, thus, be tailored to client needs, and there is existing evidence to support the use of DBT‐informed interventions for severe mental illness. We aimed to assess acceptability (uptake, completion, service‐user views of the intervention) and potential clinical impact (pre–post change in psychological distress/recovery and targeted ER processes: self‐reported ER difficulties, adaptive ER skill use). We did not measure psychotic symptoms as improvements in affective processes are more in line with service‐user priorities and may be more acceptable, recovery‐focused treatment targets (Law, Shryane, Bentall, & Morrison, 2016; Leendertse et al., 2021), and the intervention is intended to increase recovery by targeting processes implicated in associated distress and functioning rather than psychotic experiences.

## Method

### Service context

Lambeth community psychosis services are provided by South London and Maudsley National Health Service Foundation Trust. Services are for working‐age adults with established psychosis or current, persistent, distressing/disabling psychotic symptoms as the main presenting problem in the context of affective or other complex mental health conditions. We evaluated incorporating the MEG into routine care (which includes specialist care coordination, medication, and access to individual, family‐based, and group psychological therapies).

This service evaluation was approved by the local audit and evaluation committee (Local approval: PPF‐PSYCHOLO‐14‐55). Participants consented to the use of their anonymised data and basic demographic information (age, gender, and ethnicity^1^).

### Design, participants and procedure

In our local service in a single trust, a *before‐and‐after* interventional quantitative evaluation was combined with a post‐intervention qualitative focus group and individual interviews to address the evaluation aims:

1. Acceptability: uptake, completion, and service‐user’s views of the intervention (post‐session satisfaction ratings and post‐group qualitative data).
2. Potential clinical impact: pre‐ and post‐intervention self‐report measures of psychological distress, ER difficulties, and adaptive ER skill use, evaluating translation of outcomes into our local services.

Reporting of this evaluation adheres to the Strengthening the Reporting of Observational Studies in Epidemiology (STROBE) standard (von Elm et al., 2007) and the Consolidated Criteria for Reporting Qualitative Research (COREQ) (Tong, Sainsbury, & Craig, 2007) (available as supplementary appendices).

Participants were referred by community psychosis service clinicians. Exclusion criteria were broad to maximise access: insufficient English to understand assessment/therapy materials and clinical presentation precluding participation (e.g., current risk to others). Participants were asked to complete pre‐ and post‐intervention self‐report measures and provided with post‐session satisfaction forms to complete anonymously. Outcome measures were taken away to complete at home or completed during the initial assessment in the presence of a group facilitator. Only completers (attending ≥50% of sessions) were approached for post‐intervention data. Eight groups took place between September 2015 and September 2019, delivered based on clinicians’ capacity alongside other clinical work. Participants were assigned to groups following initial assessment. Each group was advertised until 6–10 participants were accepted. Fifteen individuals were approached to provide qualitative feedback (consecutive completers^2^ of the first and last groups) to assess initial and ongoing acceptability and based on availability of an interviewer external to the MEG).

### Delivery of the intervention

All groups were facilitated by a clinical psychologist and supervised by an experienced DBT clinician (CH) The lead facilitator of six groups (CL) had attended DBT skills training workshops and co‐facilitated DBT‐skills groups in a full DBT program. The other lead facilitator (JD) initially co‐facilitated with the first author. Group sessions were co‐facilitated by one or two additional professionals from the community psychosis teams (psychological therapists, occupational therapists, and psychiatric nurses). Text message reminders were provided to support attendance.

### Feasibility of local delivery: Acceptability

#### Uptake and completion

Treatment uptake was assessed by the number of participants attending ≥ one session. Completion was defined as attending ≥50% sessions.

#### Service‐user views: post‐session satisfaction ratings

The satisfaction measure (adapted from Attkisson & Zwick, 1982) comprises five questions rated on a 5‐point Likert scale evaluating usefulness, comprehensibility, interest, extent of learning, and likelihood of recommending MEG to friends/family. Higher numbers indicate greater satisfaction.

#### Service‐user views: post‐group qualitative data collection

Post‐group qualitative data were sought from participants completing the first and last groups using a semi‐structured interview schedule (see supplementary appendices) developed by the project team to reflect the evaluation aims. Interviews were conducted flexibly but structured around the following areas: reasons for attending; understanding and skills gained; impact on everyday life; un/helpful aspects; and suggested improvements. Participants were approached by phone by the interviewer and provided written informed consent to audio‐recorded interviews and anonymised data use prior to being interviewed. Interviews were conducted at the community psychosis service by a trainee or assistant psychologist external to the MEG (including TDS). Interviews lasted from 16–45 min. Two participants declined to be audio‐recorded, so field notes including verbatim quotes were taken instead, and member checks were conducted for comments and correction. Recorded data were transcribed verbatim.

### Feasibility of local delivery: Potential clinical impact

#### Pre–post outcome measures

*Psychological Distress (Clinical Outcomes in Routine Evaluation Outcome Measure,* CORE‐10; Barkham et al., 2013). The CORE‐10 is a 10‐item measure of global distress and the routine psychological therapy recovery outcome measure in our services. Scores range from 0–40; >10 indicates clinical distress (Connell & Barkham, 2007). It has robust psychometric properties and has been used in psychosis samples (Chadwick, Sambrooke, Rasch, & Davies, 2000; Durrant, Clarke, Tolland, & Wilson, 2007).

#### Self‐reported ER difficulties: (Difficulties in Emotion Regulation Scale‐16, DERS‐16; Bjureberg et al., 2016)

The DERS‐16 is informed by Gratz and Roemer’s (2004) conceptual definition of ER difficulties and assesses individual typical levels of difficulty across five domains (non‐acceptance of emotions, lack of emotional clarity, limited access to effective ER strategies, and difficulties in controlling impulsive behavior and engaging in goal‐directed behavior when distressed). Sixteen items are rated on a 5‐point scale from 1 = “almost never” to 5 = “almost always” (total score: 16–80). Higher scores reflect greater ER difficulties. It demonstrates good internal consistency, test–retest reliability, convergent and discriminant validity, and responsiveness to ER interventions in various samples, and its internal consistency and convergent validity are supported with individuals in psychosis (Lawlor, Vitoratou, Hepworth, & Jolley, 2021).

#### Adaptive ER Skill Use (DBT Ways of Coping Checklist – DBT‐WCCL; Neacsiu, Rizvi, Vitaliano, Lynch, & Linehan, 2010)

The DBT‐WCCL is a 59‐item measure of DBT skills and maladaptive, non‐DBT skills use to manage difficult situations over the past month. All items are rated from 0 = “never use” to 3 = “always use”. We used only the 38‐item DBT skills Subscale (DSS) (total score: 0–114), which demonstrates excellent internal consistency, adequate test–retest reliability, and good internal consistency across clinical samples (e.g., Brown et al., 2019; Cavicchioli et al., 2019; Stein, Hearon, Beard, Hsu, & Björgvinsson, 2016) and sensitivity to changes following DBT skills training (Neacsiu, Eberle, Kramer, Wiesmann, & Linehan, 2014; Neacsiu, Rizvi, & Linehan, 2010).

### Intervention

The MEG consists of eight 90‐min sessions (including a 5–10 min break), delivered weekly. It was developed to support emotional awareness, understanding and acceptance, and adaptive responses to emotions through teaching mindfulness, distress tolerance, and ER skills (see Table 1). Sessions were structured according to Linehan’s (2015) protocol but modified to include a recap of the preceding session to consolidate understanding and a briefer homework review. Skills and underpinning concepts were taught using everyday language, visual aids, and metaphors, and key skills were practised in each session. Space was provided to discuss psychotic experiences where relevant to skill use, with care taken not to invalidate strongly held beliefs. Simplified versions of handouts and worksheets were developed to summarise session content and encourage skill practice and generalisation. Homework completion was not required but was positively reinforced. Sessions were shorter in duration than full‐model DBT groups, and content was paced to accommodate cognitive and attentional difficulties.

**Table 1:** Summary of the managing emotions group content

Session	Focus (DBT Module)	Content
1	Mindfulness; Emotion Regulation	What emotions do for you Model for describing emotions (simplified) Mindfulness “What” and “How” skills Wise Mind
2	Distress Tolerance	STOP skill TIP skills
3	Distress Tolerance	Distraction Self‐soothing Improving the moment
4	Emotion Regulation	Ways to describe emotions
5	Emotion Regulation	Mindfulness of thoughts Check the facts
6	Emotion Regulation	Mindfulness of emotion Opposite Action
7	Emotion Regulation	PLEASE skills Accumulating positive emotions Build mastery
8	Emotion Regulation	Problem solving Cope ahead Summary of sessions

Note

STOP = Stop, Take a moment, Observe, Proceed mindfully; TIP = Temperature, Intense exercise, Paced breathing and paired muscle relaxation; PLEASE = PhysicaL health, balance Eating, avoid mood Altering substances, balance Sleep, get Exercise.

### Sample size justification

No formal sample size was calculated, but consistent with other feasibility studies, recruitment of 50 participants is sufficient to estimate the feasibility parameters. For the qualitative sample, data from 8–15 participants were anticipated to generate sufficient depth of data.

### Data analysis

Statistical analyses were carried out using SPSS software (Version 27, IBM, 2020). Pre‐and post‐group scores on the clinical outcome measures were compared using paired‐sample *t*‐tests. Mean differences (95% CI, *p* values) and effect sizes (Cohen’s *d*) were also reported accordingly. Where appropriate, values were reverse‐scored to ensure all outcomes indicated a positive outcome in a consistent manner. Associations between pre–post changes in outcomes were explored using Pearson’s correlation coefficients.

Qualitative data were analyzed using a process of inductive thematic analysis as described by Braun and Clarke (2006). This method was chosen as it provides a flexible approach to developing a rich account of patterns and common themes in relation to participant experiences. The analysis was iterative and involved six phases: (1) familiarisation with the dataset; (2) generating codes across the dataset; (3) constructing themes; (4) reviewing themes; (5) defining and naming themes, and (6) writing up the themes. A critical realist perspective was adopted which assumes that the ways in which participants talk about their experiences provide access to their ‘reality’, *and* representations of reality are shaped by a range of factors. Analyses were conducted by the first author (a clinical psychologist trained in qualitative analysis), who was mindful of the potential impact of their positioning and background and used reflexivity to ensure rigor. The first author was involved in developing and cofacilitating the MEG and had knowledge of the participant group and relevant research and theory. The coding and themes were shared with another author (CLB, qualitative researcher, external to MEG) to provide an additional perspective to enhance the analyses. Qualitative data were managed in Microsoft Word.

## Results

### Demographic and clinical characteristics

Table 2 shows demographic and clinical characteristics of the included participants.

**Table 2:** Participant demographic and clinical information

	Total sample (*n* = 75)	Completers (*n* = 48)
Mean age in years (SD, range)	44 (11, 26–66)	43 (11, 26–64)
Gender		
Male	41 (55%)	22 (46%)
Female	34 (45%)	26 (54%)
Ethnic group		
BME	42 (56%)	25 (52%)
Non‐BME	33 (44%)	23 (48%)
Diagnosis		
Schizophrenia	35 (47%)	23 (48%)
Schizoaffective disorder	16 (21%)	11 (23%)
Bipolar affective disorder	12 (19%)	6 (13%)
Persistent delusional disorder	4 (5%)	4 (8%)
Unspecified nonorganic psychosis	4 (5%)	2 (4%)
Psychotic depression	2 (3%)	0 (0%)
Acute and transient psychotic disorder	1 (1%)	1 (0.2%)
Other nonorganic psychotic disorder	1 (1%)	1 (.02%)

Note

BME = Black/ Minority Ethnic group; DERS‐16 = Difficulties in Emotion Regulation Scale‐16 item version; DSS = Dialectical Behavior Therapy Ways of Coping Checklist‐ DBT Skills Subscale; CORE‐10 = Clinical Outcomes in Routine Evaluation – 10 item version; SD = Standard Deviation.

### Feasibility of local delivery: Acceptability

#### Uptake and completion

Of the 75 participants assessed and offered the intervention, 61 attended at least one session (81.3%), of whom 48 (78.7%) completed the intervention. There was little difference between completers and non‐completers (dropouts and non‐attenders) in relation to age, gender, ethnicity, or pre‐treatment outcome measures. Mean attendance was 5.34 of the eight offered sessions (*SD* = 2.97). Of the 48 completers, 27% (13) attended every session, 27% (13) attended seven, 19% (9) attended six, 14.5% (7) attended five, and 12.5% (6) attended four sessions. Of the 11 non‐completers, 54% attended one session (6), 18% attended two (2), and 27% attended three (3).

#### Service‐user views: post‐session satisfaction ratings

Most participants completed post‐session feedback forms (total forms = 297; completion rate = 89.7%). Ratings of usefulness (*M* = 4.34, *SD* = 0.66), comprehensibility (*M* = 4.29, *SD* = 0.72), interest (*M* = 0.25, *SD* = 0.72), and extent of learning (*M* = 4.25, *SD* = 0.66) indicated high satisfaction. Furthermore, 94% of participants reported that they would recommend MEG to friends and family (extremely likely: *n* = 147; likely: *n* = 119).

#### Service‐user views: post‐group qualitative results

Twelve participants agreed to provide qualitative feedback (three did not reply). Participant demographic and clinical information is available as a supplementary appendix. Five overarching themes were identified in the data: (1) Feeling controlled by emotions; (2) Other people feel the same way; (3) Learning to tune into and understand emotions; (4) I can change my emotions; (5) Continuing to use skills in everyday life. The first theme describes participant experiences of ER difficulties prior to MEG, themes 2–4 focus on the impact of MEG, and the final theme focuses on the implications for the future.

### Theme one: Emotions controlled me

Participants reported difficulties managing their emotions prior to attending the MEG. Emotions were described as intense, overwhelming, and a barrier to functioning well in everyday life. They emphasised wanting “different skills” (P10) to lessen the negative impact of their emotions.

I asked if there were any groups which can help me come out of my emotions… mostly it’s the voices and things that happened in the past… it’s like somebody has stabbed you and the wound hasn’t healed (P7)

(I wanted) techniques to get through high, intense emotion… Emotions were getting to the point of becoming overwhelming… not being able to function properly through day‐to‐day normal activities (P8)

I was struggling with controlling my emotions… I didn’t have a plan, it just used to get out of control. I used to start crying… (and had) suicidal thoughts, feeling, hopeless, worthless (P9)

### Theme two: Validation through shared experiences

All participants valued being part of a group and learning from one another. Group discussions helped individuals see their emotions as valid and normal and realise that “*other people are in a similar situation*” (P12). One participant felt that group facilitators sharing their own experiences helped convey that everyone needs skills and can experience difficulties in managing their emotions. However, another participant reflected that responding differently to a group exercise led them to conclude that their emotional reaction was abnormal. Being part of a group may, thus, add to a sense of difference if individuals equate normal emotional reactions to reacting similarly to other people.

It’s just nice in a group because you don’t feel so on your own (or) stupid for feeling that way… It helped (me) recognise … all emotions are normal (P1)

The focus wasn’t on mental health, it was on emotions… It felt more normal and less alienated. (Facilitators discussed applying) the techniques themselves, even though they haven’t had a mental health issue (P10)

Everybody found it quite amusing, but the music made me quite anxious … I felt like the odd one out (P5)

### Theme three: Learning to tune into and understand emotions

Participants reported gaining understanding of “*how emotions work*” (P6) and how to attend to them. Experiential exercises: “*examples, practical games, and visual stuff… rather than just talking*” (P2) appeared to facilitate this. This included the realisation that emotions “*will pass*” (P10) and “*you don’t have to get rid of (emotions), it’s just managing them*” (P1). Some participants also gained insights into past experiences of emotional distress including their mental health difficulties.

We all had a card with a different emotion and acting it out made it a bit more real and made you more conscious of what an emotion is and what it can feel like (P5)

It explained to me like why I was having those feelings and … why sometimes our emotions get out of control… Because of the group, I’ve noticed my emotions a lot better. I’m able to catch them before they get too extreme (P9)

Looking back on past emotions (I was) able to understand why things got to the point that they got to, and I had to join services in the first place (P8)

### Theme four: I can change my emotions

Participants reported gaining confidence that they can change unwanted or persistent emotions and pursue valued activities. Some emphasised the helpfulness of having a range of tools to meet different needs.

When I feel stressed, I start trying some techniques… Like when I feel anxious, I cook something good, so that it can change my emotions…or go to the park (P4)

(I liked) the deep breathing techniques and there was one where you hold your breath, dip your face in water. I found that really, really good… slowing my thought process down and calming me … Now I’m able to cope and be productive during my days, rather than getting bogged down by it all and finding myself feeling stuck (P8)

It’s putting it into a category to say ok this is what mindfulness is, and when I am in this situation, I know that this is what I can do… and it will help (P6)

### Theme five: Continuing to use skills in everyday life

All participants reported planning to continue to apply skills gained. Written resources were emphasised as being very helpful; however, some participants felt that additional information including how to apply skills to specific situations in their lives would be useful.

It’s like a toolbox … so hopefully when things come up you have more things at your disposal (P1)

The good thing about the group is you can look back on the worksheets and see how they can help you (P3)

Maybe if there were… written down situations and then you say how you would normally react and then do a way around that… I guess it’s just judging each emotion, each situation differently (P8)

### Feasibility of local delivery: Potential clinical impact

#### Pre–post outcome measures

Paired outcome measures were obtained for 39 (81%) completers. Missing data were due to some participants declining or omitting to complete all measures. Table 3 presents descriptive statistics for all measures and analyses of pre‐ and post‐intervention effects. Paired‐sample t‐tests found significant reductions in psychological distress (improved recovery) with large effects (mean difference (MD) = 3.7, 95% CI = 0.2–1.2, *d* = 0.7). There were also significant reductions in overall specific ER difficulties, with most effects moderate–large in magnitude (Total difficulties: MD = 9, 95% CI = 0.3–1, *d* = 0.7; Clarity: MD = 0.7, 95% CI = 0–0.7; *d* = 0.3; Goals: MD = 1.9, 95% CI = 0‐2–0.9; *d* = 0.5; Impulse: MD = 1.8, 95% CI = 0.3–1, *d* = 0.6; Strategies: MD = 3.1, 95% CI = 0.3–1, *d* = 0.6; Nonacceptance: MD = 1.5, 95% CI = 0.2–0.9, *d* = 0.5). Adaptive ER skill use significantly increased, with large effects (mean difference = 11.3, 95% CI = 0.3–1.1, *d* = 0.7).

**Table 3:** Pre‐post intervention clinical outcomes (completer analyses)

Measure (min‐max score)	Pre‐Group				Post‐Group				Comparison		
	α	*n*	Mean (SD)	Median (min‐max)	α	*n*	Mean (SD)	Median (min‐max)	Mean diff (95% CI)	*p* value	ES (d)
DERS‐16 Clarity (2–10)	0.77	67	5.01 (2)	5 (2–10)	0.86	39	4.18 (1.7)	4 (2–10)	0.7 (0,0.7)	0.040	0.3
DERS‐16 Goals (3–15)	0.81	67	9.51 (3.4)	10 (4–15)	0.89	39	7.77 (3.1)	7 (3–15)	1.9 (0.2,0.9)	0.002	0.5
DERS‐16 Impulse (3–15)	0.84	67	7.36 (3.7)	7 (3–15)	0.89	39	4.87 (2.7)	4 (3–14)	1.8 (0.3,1)	<0.001	0.6
DERS‐16 Strategies (5–25)	0.76	67	14.09 (4.6)	13 (6–24)	0.63	39	11.13 (4.2)	10 (5–23)	3.1 (0.3,1)	<0.001	0.6
DERS‐16 Non‐Acceptance (3–15)	0.81	67	8.18 (3.5)	8 (3–15)	0.84	39	6.59 (2.8)	6 (3–12)	1.5 (0.2,0.9)	0.002	0.5
DERS‐16 Total (16–80)	0.92	67	44.15 (14)	41 (22–76)	0.94	39	34.54 (12.2)	30 (17–63)	9 (0.3,1)	<0.001	0.7
DSS (0–114)	0.91	57	67.67 (19.7)	68 (10–114)	0.74	31	80.71 (13.7)	81 (53–108)	11.3^a^ (0.3, 1.1)	0.001	0.7
CORE‐10 (0–40)	0.81	54	15.15 (7.2)	13 (5–34)	0.91	26	12.06 (9.1)	10 (1–31)	3.7 (0.2,1.2)	0.003	0.7

Note

DERS‐16 = Difficulties in Emotion Regulation Scale‐16 item version; DSS = Dialectical Behavior Therapy Ways of Coping Checklist‐ DBT Skills Subscale; CORE‐10 = Clinical Outcomes in Routine Evaluation – 10 item version; SD = Standard Deviation; CI Confidence Intervals.

^a^ Mean value subtracted from 1.

Finally, as shown in Table 4, pre–post DSS changes were negatively correlated with changes in overall ER difficulties and difficulties in relation to strategies, non‐acceptance of emotion and pursuit of goals (all *p* values < .001), and there was a trend‐level association with changes in impulse control (*p* = 0.072). Changes in distress were only correlated with changes in difficulties in pursuing goals. Differences in the DERS‐16 scores were correlated with moderately strong–strong correlation coefficients.

**Table 4:** Correlations between changes in scores on outcome measures (Time 2‐Time 1)

	DSS	CORE‐10	DERS‐16 clarity	DERS‐16 goals	DERS‐16 impulse	DERS‐16 strategies	DERS‐16 non‐acceptance
DERS‐16 Clarity	−0.27 (0.177)	0.22 (0.333)					
DERS‐16 Goals	−0.59 (0.002)	0.57 (0.007)	0.51 (0.001)				
DERS‐16 Impulse	−0.36 (0.072)	0.32 (0.163)	0.46 (0.004)	0.58 (<0.001)			
DERS‐16 Strategies	−0.68 (<0.001)	0.2 (0.384)	0.59 (<0.001)	0.68 (<0.001)	0.69 (<0.001)		
DERS‐16 Non‐Acceptance	−0.61 (0.001)	0.26 (0.254)	0.47 (0.003)	0.51 (0.001)	0.43 (0.009)	0.64 (<0.001)	
DERS‐16 total	−0.62 (0.001)	0.38 (0.088)	0.71 (<0.001)	0.83 (<0.001)	0.79 (<0.001)	0.92 (<0.001)	0.75 (<0.001)

Note

DERS‐16 = Difficulties in Emotion Regulation Scale‐16 item version; DSS = Dialectical Behavior Therapy Ways of Coping Checklist‐ DBT Skills Subscale; CORE‐10 = Clinical Outcomes in Routine Evaluation – 10 item version.

## Discussion

This evaluation was conducted in routine clinical practice and found that a brief DBT‐informed group for individuals with established psychosis was feasible and acceptable (uptake, completion, service‐users’ views) and associated with potential clinical impact (pre‐post outcome measures). Rates of uptake were around 80%, with nearly 80% of those completing, comparing favorably to other group interventions for psychosis (Sedwick, Hardy, Newbery, & Cella, 2021). Service users reported high satisfaction and valued outcomes, including increased emotional awareness, understanding and acceptance, self‐efficacy in relation to managing emotions, and pursuit of valued activities. Self‐reported psychological distress, ER difficulties, and adaptive ER skill use all significantly improved, suggesting that positive outcomes from research translate into clinical improvement in our local diverse inner‐city context.

Pre–post changes in self‐reported ER difficulties are consistent with previous work (Ryan et al., 2021; Silva et al., 2020) and effect sizes in our local setting compare favorably those reported by research (Ryan et al., 2021; Valentine et al., 2020). Generalisation of adaptive ER skills into everyday life is considered a primary mechanism of change for emotional dysregulation, mediating treatment effects across clinical populations (Neacsiu et al., 2014; Rudge, Feigenbaum, & Fonagy, 2020; Southward, Sauer‐Zavala, & Cheavens, 2021). In the present evaluation, participants reported significantly increased adaptive ER skill use at post‐intervention which was associated with improvements in self‐reported ER difficulties. However, extent of skill use beyond the MEG is unknown. Furthermore, qualitative accounts suggested that additional support may be needed to support transfer to everyday life. Finally, in line with the qualitative findings, self‐reported improvements in distress were related to self‐reported improvements in pursuing goals in the context of negative emotion.

### Strengths and limitations

Our findings support the feasibility of local delivery in an inner‐city context and provide depth and detail about how participants experienced the intervention and evidence of potential clinical impact. Several limitations should be noted. This was a pre–post service evaluation in a small sample, without a control group. Access to other interventions was unrestricted; positive outcomes cannot be reliably attributed to the MEG. The evaluation took place over a four‐year period, matching clinician time and capacity. Other service‐related changes may have occurred within that time, influencing potential clinical impact data. Outcome data were sought only from completers and qualitative feedback only from the first and last groups. Some participants completed outcome measures in the presence of a facilitator, which may have affected their responses. Reasons for non‐attendance or drop‐out were not recorded. The findings are specific to those who participated and provided data in our local services. Finally, treatment fidelity was not formally assessed (Leichsenring et al., 2011).

### Research recommendations for the development of service‐based implementation

Several areas warrant further research evaluation. A key question raised by the evaluation is how to best support participants to apply adaptive ER skills both during (e.g., *via* phone coaching, supplementary individual sessions) and following DBT‐informed interventions (e.g., *via* digital technology aids, follow‐up sessions) to maximise outcomes. Second, more research is needed on the acceptability of DBT‐informed interventions in psychosis, including the views of dropouts and non‐attenders Third, although findings to date are promising, controlled studies are needed to compare DBT‐informed interventions to other active treatments, evaluate impact on a wider range of outcomes (e.g., psychotic symptoms, recovery indices), and determine the durability of positive outcomes to clarify the potential benefits of implementation. Involving service‐users in refinement and evaluation work (e.g., optimising its format, duration, content, and determining meaningful outcomes) will benefit future research. Finally, research utilising multi‐modal and comprehensive assessment of ER difficulties and skill use is needed both to clarify the treatment needs and priorities of individuals with psychosis and the processes associated with positive outcomes.

### Conclusions

We found that a brief DBT‐informed intervention was feasible for delivery in our local community psychosis setting. Satisfaction and attendance levels were high, and service‐users reported valued improvements in understanding emotions and feeling able to manage them. Research is now needed to investigate the feasibility of a controlled trial and further refine the intervention to best meet the needs and priorities of individuals with psychosis.

## Data availability statement

Data available on request from the authors.

## Author contributions

**Ben Carter** (Methodology; Supervision; Writing – review & editing) **Clair Le Boutillier** (Supervision; Writing – review & editing) **Suzanne Jolley** (Conceptualization; Methodology; Supervision; Writing – review & editing) **Claire Hepworth** (Conceptualization; Methodology; Supervision; Writing – review & editing) **Tanisha De Souza** (Investigation; Writing – review & editing) **Silia Vitoratou** (Data curation; Formal analysis; Writing – review & editing) **Caroline Lawlor**, BSc, DClin Psy, PGDip (Conceptualization; Formal analysis; Investigation; Writing – original draft) **Ben Cooper** (Writing – review & editing) **James Duffy** (Writing – review & editing).

## Conflicts of interest

All authors declare no conflict of interest.

## Supporting information


**Appendix S1**. STROBE statement.

**Appendix S2**. Consolidated criteria for reporting qualitative studies (COREQ): 32‐item checklist.

**Appendix S3**. Qualitative sample information.

**Appendix S4**. Qualitative interview topic guide.

**Appendix S5**. Qualitative interview topic guide.

## Footnotes

^1^
Data were extracted from the clinical record. Ethnicity data are collected based on the ethnic group categories defined by the Census for England and currently form the national standard for reporting in the NHS.

^2^
In line with similar studies (e.g., Khoury et al., 2015; Ryan et al., 2021), completion (receiving an intervention “dose”) was defined as attending ≥50% sessions
