# Supplementary material for: Managing emotions in psychosis: Evaluation of a brief DBT‐informed skills group for individuals with psychosis in routine community services
Source: Br J Clin Psychol. 2022 Feb 7;61(3):735–56. doi: 10.1111/bjc.12359 (PMC9543194; doi:10.1111/bjc.12359)
Supplement: Supplementary file 3 — Appendix S3. Qualitative sample information. [file BJC-61-735-s003.docx]

**Supplementary information C: Qualitative sample information**

| Participant number | Gender | Age | Ethnicity | Diagnosis | # sessions attended |
| --- | --- | --- | --- | --- | --- |
| P1 | Female | 30 | Non BME | Schizoaffective disorder | 4 |
| P2 | Male | 40 | Non BME | Bipolar affective disorder | 7 |
| P3 | Female | 34 | BME | Schizophrenia | 7 |
| P4 | Female | 41 | Non BME | Delusional disorder | 6 |
| P5 | Female | 45 | BME | Schizoaffective disorder | 6 |
| P6 | Female | 43 | BME | Schizophrenia | 4 |
| P7 | Female | 64 | BME | Schizoaffective disorder | 6 |
| P8 | Male | 26 | Non BME | Schizophrenia | 5 |
| P9 | Female | 27 | BME | Schizophrenia | 4 |
| P10 | Female | 39 | Non BME | Bipolar affective disorder | 7 |

Key: BME: Black/ Minority Ethnic group
