# Supplementary material for: Managing emotions in psychosis: Evaluation of a brief DBT‐informed skills group for individuals with psychosis in routine community services
Source: Br J Clin Psychol. 2022 Feb 7;61(3):735–56. doi: 10.1111/bjc.12359 (PMC9543194; doi:10.1111/bjc.12359)
Supplement: Supplementary file 4 — Appendix S4. Qualitative interview topic guide. [file BJC-61-735-s004.docx]

**Supplementary Information D: Qualitative Interview Topic Guide**

1. Can you tell me a bit about why you decided to attend the group?

Probes:

- What were your expectations before you started?
- Did you have any ideas about what the group might be like?
- Any concerns/worries/hopes?
- How well did you feel you managed your emotions before the group?

1. What was your overall experience of the group?

Probes:

- Were there particular things you liked? What was good about them?
- Anything you found less helpful or did not like?
- What do you think would have been better/more helpful?
- Did the group meet your expectations?
- What was it like being in a group?
- What did you like most/least about the group?
- How did you feel about the length of the sessions?
- What did you think of the session materials?
- How did you find the facilities?

1. We’re interested in knowing whether people noticed any changes after starting the group. What, if anything, did you learn in the group?

Probes:

- Is there anything different about how you understand and manage your emotions since starting the group?
- Did you gain any useful skills, strategies or information?
- Which skills did you particularly like? Which did you find less helpful?
- Have you tried out any skills since starting the group? If yes, what strategies did you use? Can you think of an example where you used that strategy? If no, what got in the way?
- How easy or difficult was it to try the skills learnt in between sessions? What helped with this/what could have made it easier?
- Since attending the group have you been more able to do things that are important to you?

1. Is there anything else that could be improved if the group were to run again?

Probes:

- Length/number of sessions, size of group, the way the group was run, skills covered, practical exercises etc. Anything else?

1. What would you tell someone who is considering attending the group but is unsure about coming and wants to know what it’s like?
2. Do you have anything more to add to any of the topics we've covered or anything else that you think is relevant to what we’ve talked about?
